# Supplementary figures and images for: Cep131-Cep162 and Cby-Fam92 complexes cooperatively maintain Cep290 at the basal body and contribute to ciliogenesis initiation
Source: PLoS Biol. 2024 Mar 5;22(3):e3002330. doi: 10.1371/journal.pbio.3002330 (PMC10914257; doi:10.1371/journal.pbio.3002330)

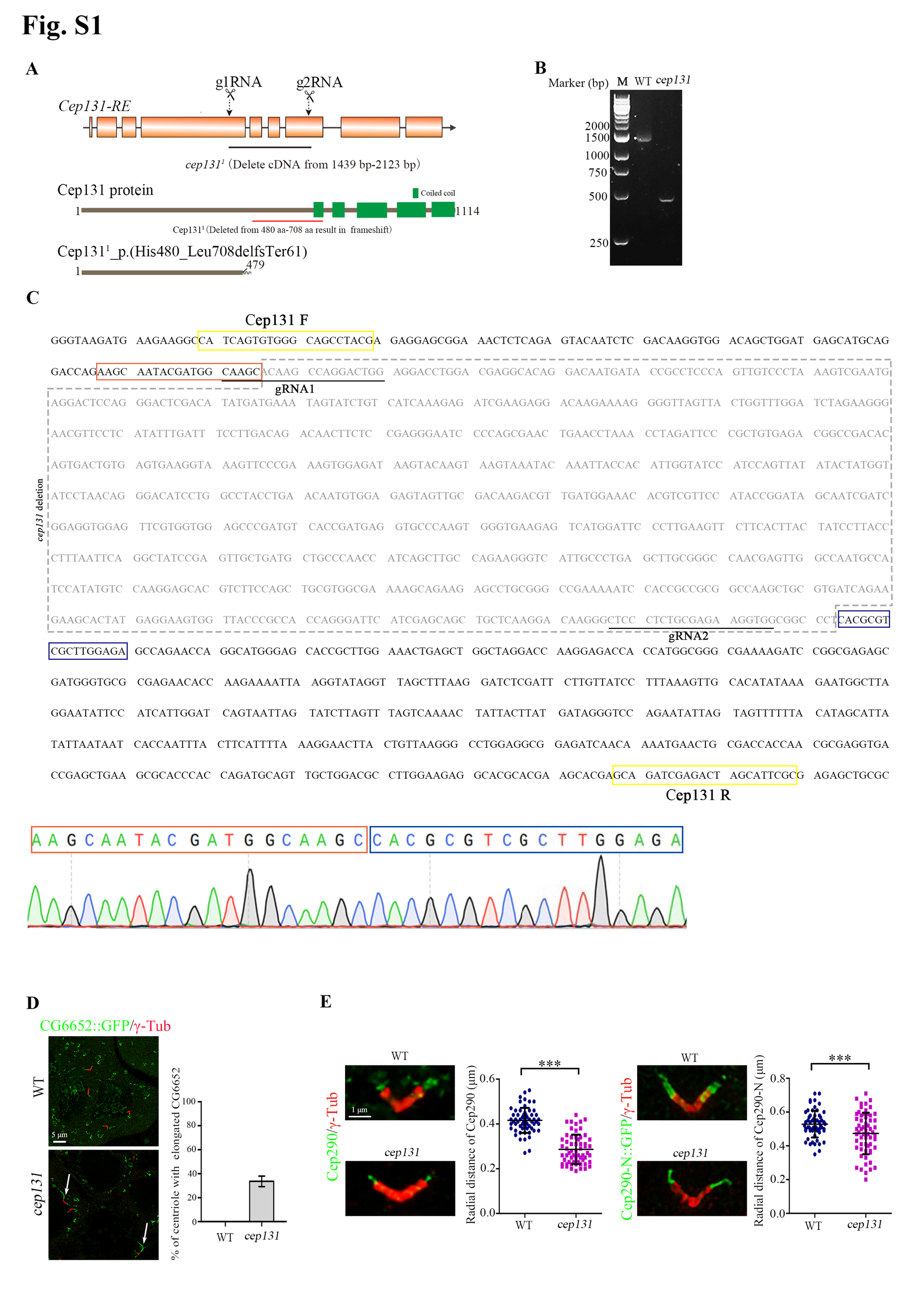

Supplement: S1 Fig — (A) Diagram showing of the generation of cep131 mutants. Schematics show the genomic (upper panel) and protein (lower panel) structures of Cep131, along with the predict protein product of Cep1311 mutant (Cep1311_p.(His480_Leu708delfsTer61)). Two arrows represent gRNA target sites. cep1311, a frameshift line, has a deletion in cDNA from nt 1439 to 2123, resulting in a reading frame shift and C-terminus loss. (B) Genotyping of cep131 mutants using PCR. The PCR amplification products were 1471 bp long for w1118 and 603 bp long for cep131 flies. (C) Sequence confirmation of the deletion in cep131 mutant. Primers used for sequence are marked with orange frames. The locations of 2 gRNAs used for mutant generation are underlined with black. Red and blue frames label the boundary of deletion cep131 in mutant. (D) Immunostaining of CG6652 in spermatocyte cilia of WT or cep1311 testis. CG6652 (green) marks the ciliary axoneme. In cep1311, a few centrioles have over elongated CG6652 signals (arrows). Centriole/basal body is marked with γ-Tubulin (red). Scale bars, 5 μm. (E) Quantification of the radial distance of Cep290 or Cep290-N. In cep131 mutants, the radial distance of Cep290 or Cep290-N were significantly reduced compared to WT. The error bars represent the mean ± SD, n = 60. The data underlying this figure can be found in S1 Data. (TIF) [file pbio.3002330.s001.tif]

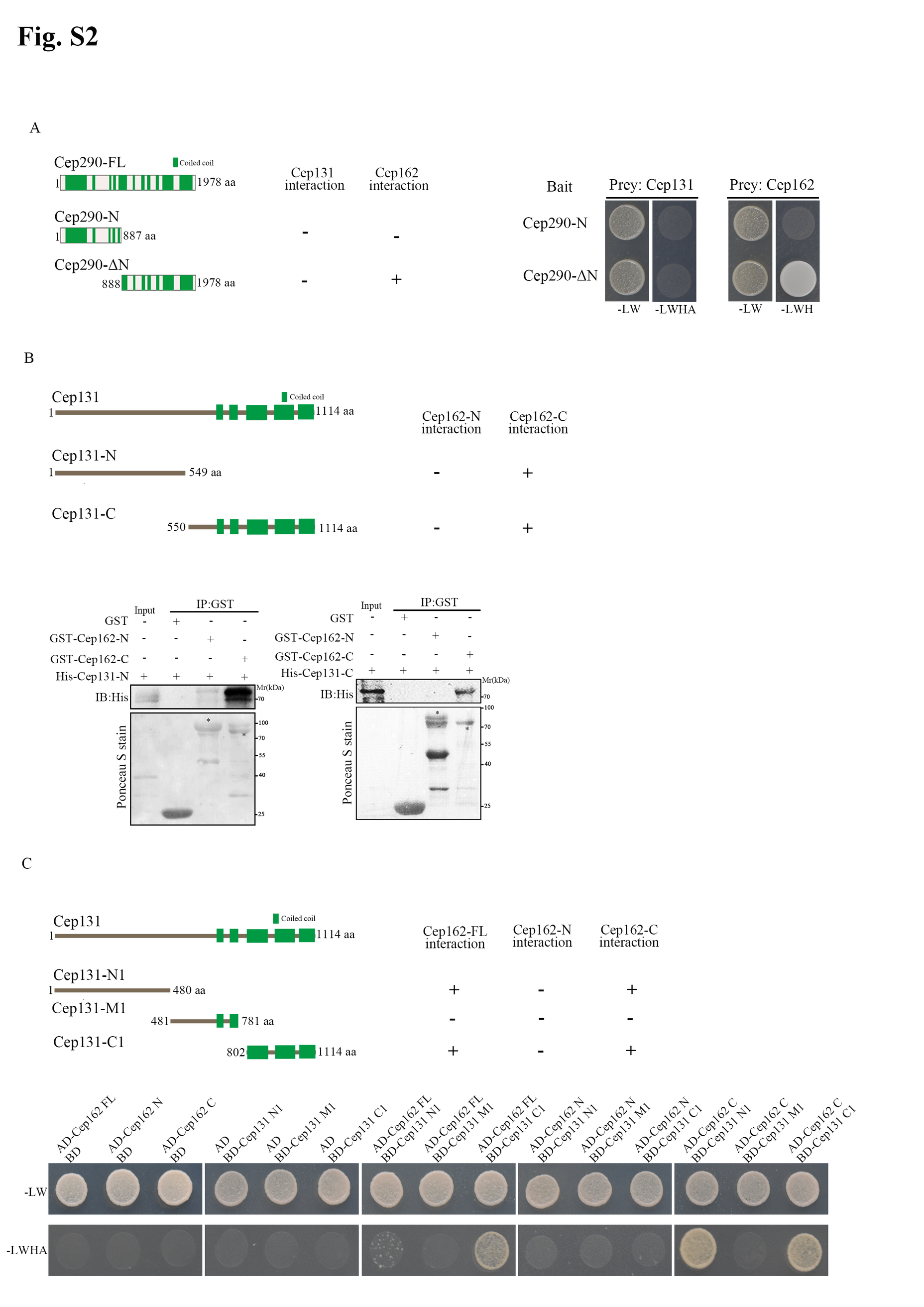

Supplement: S2 Fig — (A) In Y2H assay, Cep290 interacts with Cep162 (CG42699), but not Cep131 in Drosophila. LW: Selective media SD-Leu-Trp plates. LWH: Selective media SD-Leu-Trp-His plates. LWHA: SD-Ade-Leu-Trp-His plates. (B) The GST pull-down assay confirmed the direct interaction between Cep131 and Cep162 in vitro. GST, GST-Cep162-N, and GST-Cep162-C recombinant proteins were pulled down with His-Cep131-N or His-Cep131-C proteins. (C) In Y2H assay, Cep162 interacts with Cep131-N1 and Cep131-C1, but not Cep131-M1. LW: Selective media SD-Leu-Trp plates. LWH: Selective media SD-Leu-Trp-His plates. LWHA: SD-Ade-Leu-Trp-His plates. (TIF) [file pbio.3002330.s002.tif]

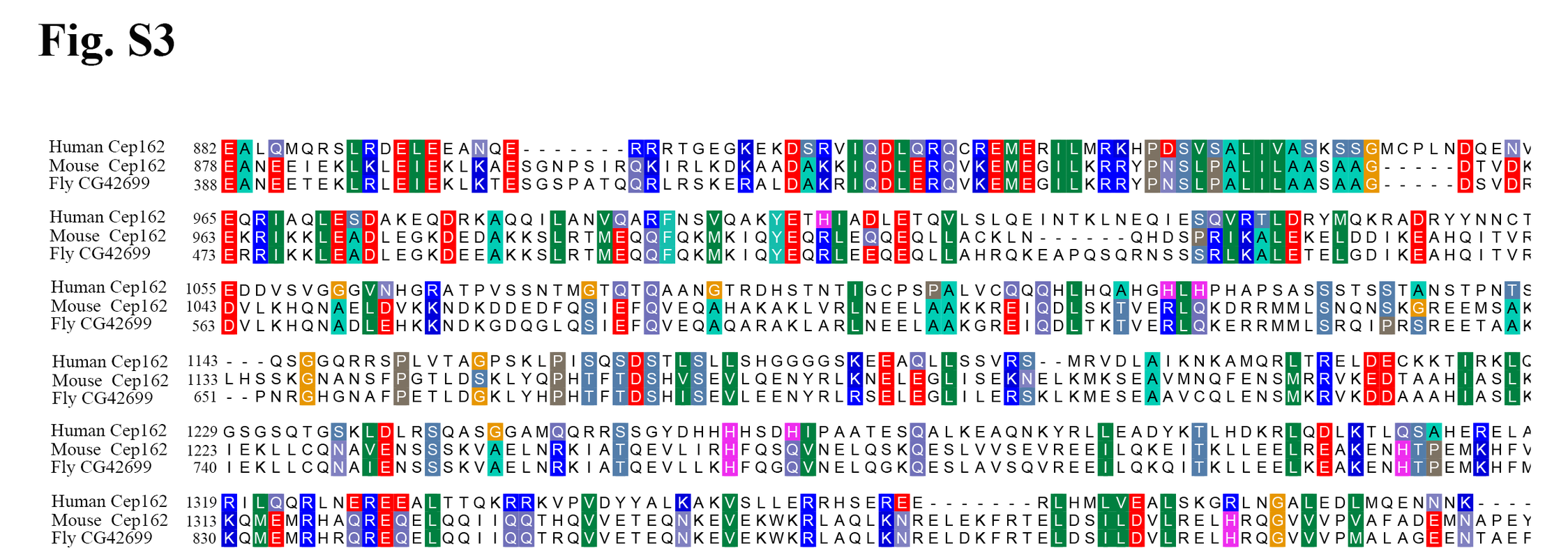

Supplement: S3 Fig — CG42699 is the only one significant alignment sequence when searching for homology of human or mice Cep162 protein in Drosophila melanogaster. (TIF) [file pbio.3002330.s003.tif]

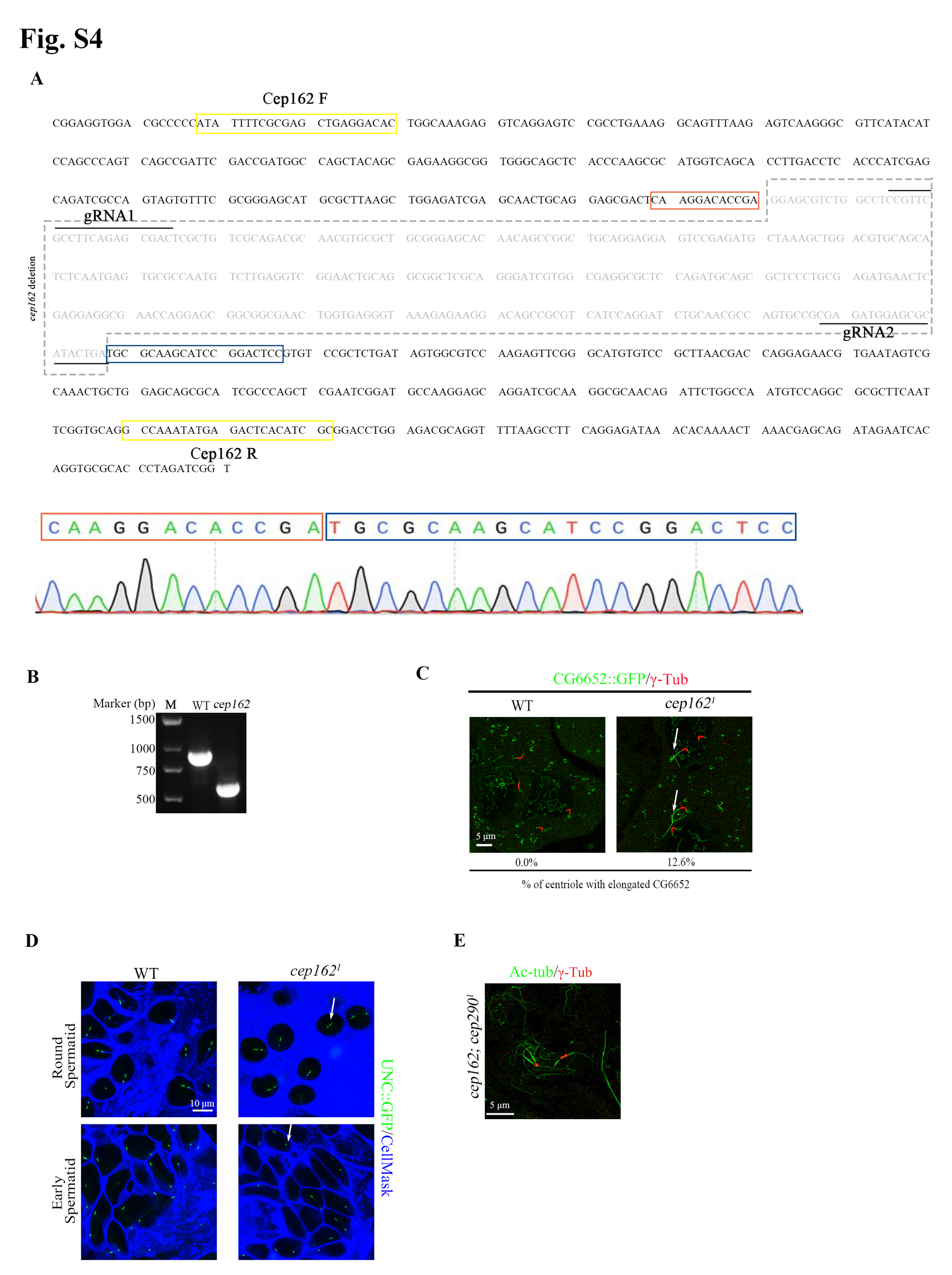

Supplement: S4 Fig — (A) Sequence confirmation of the deletion in cep162 mutant. Primers used for sequence are marked with orange frames. The locations of 2 gRNAs used for mutant generation are underlined with black. Red and blue frames label the boundary of deletion cep162 in mutant. (B) Genotyping of cep162 flies using PCR. The amplification products were 881 bp long for WT and 555 bp long for mutants. (C) Immunostaining of CG6652 in spermatocyte cilia of WT or cep1621 testis. CG6652 (green) marks the ciliary axoneme. In cep1621, a few centrioles have over elongated CG6652 signals (arrows). (D) Live imaging of the connection between the ciliary cap and the plasma membrane in WT flies and cep162 mutants. The plasma membrane (PM) was labeled with CellMask, and the BBs were marked by UNC-GFP. Defective connection between the BBs and the membrane was observed in some spermatids of cep1311 mutant (arrows). (E) Spermatocytes showing abnormal acetylated-tubulin extensions in centriole/BBs in cep162; cep2901 mutants. Scale bars, 5 μm (C, E), 10 μm (D). (TIF) [file pbio.3002330.s004.tif]

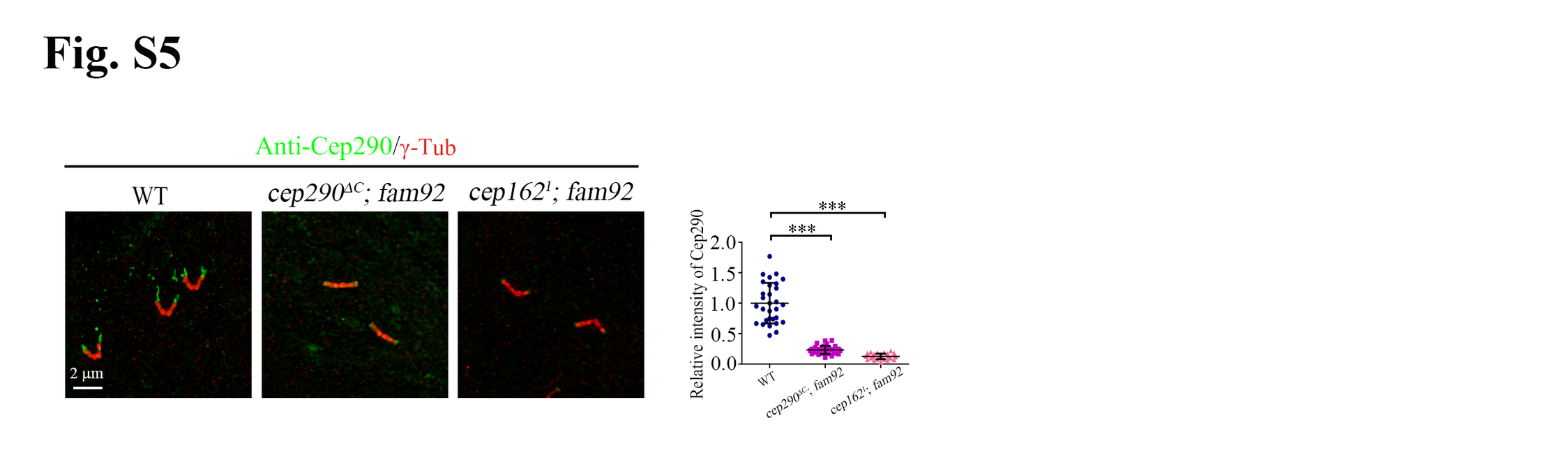

Supplement: S5 Fig — Cep290 are absent from the TZ in the spermatocyte of cep162; fam92 or fam92; cep290ΔC mutants. The BB was labeled with γ-Tubulin (red). The error bars represent the mean ± SD, n = 30. Scale bars, 2 μm. The data underlying this figure can be found in S1 Data. (TIF) [file pbio.3002330.s005.tif]

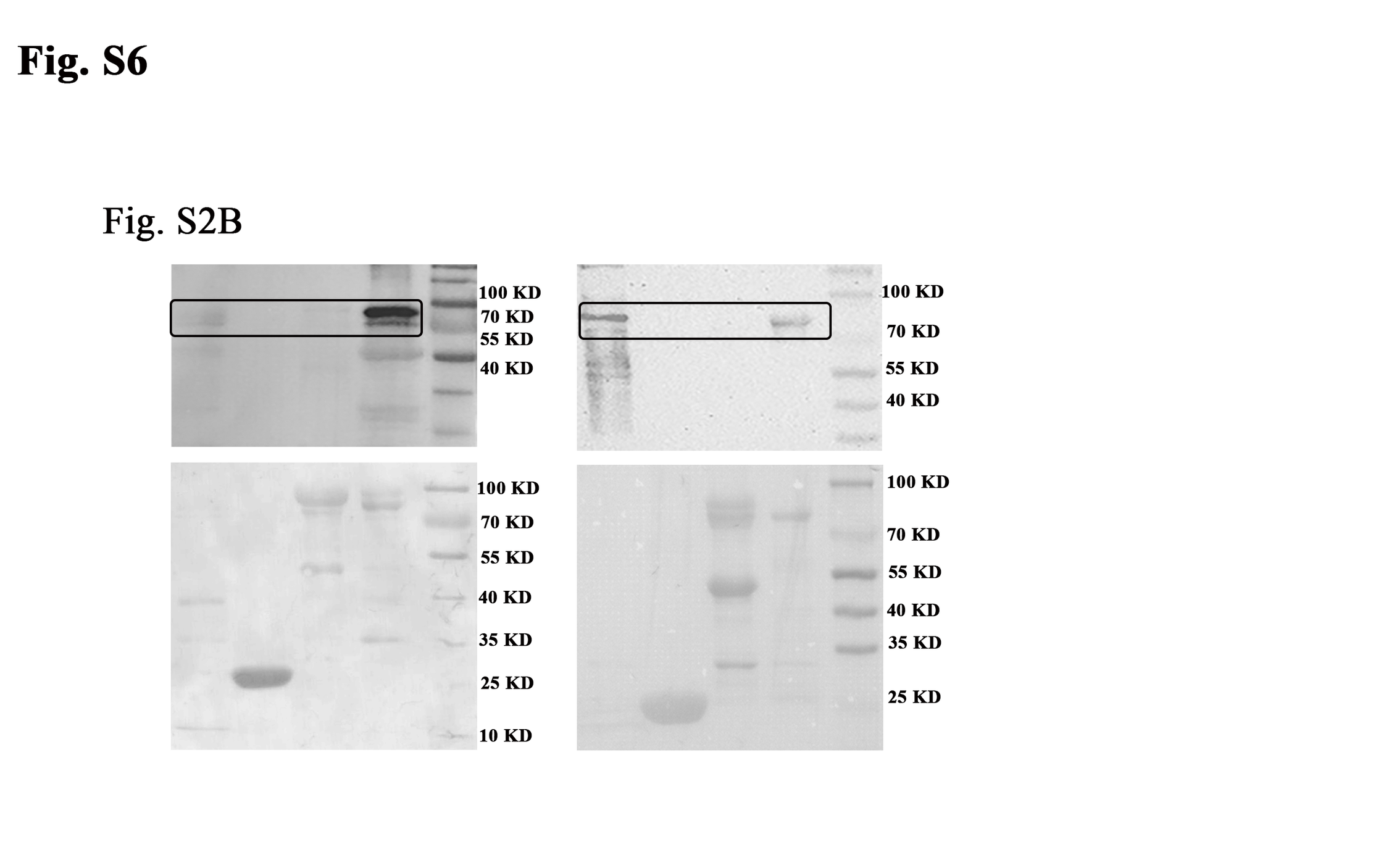

Supplement: S1 Raw Image — (TIF) [file pbio.3002330.s006.tif]
