# Supplementary material for: Cep131-Cep162 and Cby-Fam92 complexes cooperatively maintain Cep290 at the basal body and contribute to ciliogenesis initiation
Source: PLoS Biol. 2024 Mar 5;22(3):e3002330. doi: 10.1371/journal.pbio.3002330 (PMC10914257; doi:10.1371/journal.pbio.3002330)
Supplement: Attachment — Submitted filename: Response to Reviewers.docx [file pbio.3002330.s008.docx]

**Rev. 1:**
This paper by Wu et al. discusses the regulation of CEP290 localization during ciliogenesis in Drosophila. The authors demonstrate that the Cep131-Cep162 module near the axoneme and the Cby-Fam92 module at the membrane cooperate to control the basal body localization of Cep290, facilitating transition zone assembly during the initiation of ciliogenesis. Previous research (Vieillard et al., 2016) has already shown that Cep131 and Cby collaborate to recruit Cep290 and build the transition zone. However, this study adds a novel contribution by highlighting the essential role of Cep162, which bridges Cep131 and the C-terminus of Cep290 in transition zone formation. Additionally, the authors provide evidence that Dzip1-Cby-Fam92 interacts with the N-terminus of Cep290, playing a role in Cep290 recruitment. In summary, the paper proposes a model in which three central molecular pathway modules—Cep131-Cep162, Cep290, and Dzip1-Cby-Fam92—cooperatively regulate the initiation of cilium assembly. This work contributes to our understanding of the critical role of Cep290 in ciliogenesis and its regulation by other ciliogenesis modules, shedding light on related ciliopathies. **I do have some questions/concerns that should be addressed.**

We would like to thank Rev.1 for the valuable comments. Kindly find our responses addressing all of her/his concerns below.

**Major Points:**
**1.** In their previous paper, the authors showed that Cep290 acts upstream of Dzip1 and Cby (Wu et al., 2020. While Dzip1 depletion abolishes Cby localization at the TZ, but the recruitment of Cep290 was not affected. In this study, they conclude that the Dzip1-Cby-Fam92 module controls the basal body localization of Cep290. Thus, the relationship between Cep290 and the Cby-Fam92 module during ciliogenesis requires further clarification.

**Answer:** We thank the reviewer for this critical question. In this study, we illustrated the crucial role of the Cby-Fam92 module in the basal body localization of **the N-terminus of Cep290**. As the basal body localization of Cep290 is cooperatively maintained by both its N-terminus close to the membrane and C-terminus near the axoneme, the deletion of the Dzip1-Cby-Fam92 module does not disrupt the targeting of Cep290 to the basal body, instead, Cep290 could be directed to the basal body by its C-terminus through interaction with the Cep131-Cep162 module. As depicted in Figure 6C, in *cby* single mutant, endogenous Cep290 can still be recruited to the basal body; however, the C-terminus-deleted Cep290 (*cep290^ΔC^*) loses its signal at the basal body in *cby* single mutants.

In our proposed model (Fig. 7 A and B), Cep131-Cep162 module recruits Cep290, facilitating the binding of Cep290-C to the axoneme; and the N-terminus of Cep290 recruits Dzip1-Cby-Fam92 module, initiating early ciliary membrane formation and ciliary bud formation. Conversely, Cby-Fam92-mediated ciliary membrane formation exhibits a positive feedback effect, enhancing the association of Cep290 N-terminus with the ciliary membrane. In other words, Cep290 recruits the Dzip1-Cby-Fam92 module, and this module, in turn, positively influences the localization of the N-terminus of Cep290 at the basal body.

**2.** Previous work conducted in human cells has demonstrated that the localization of CEP290 at centriole distal ends is independent of CEP162, whereas CEP162 primarily recruits CEP290 to microtubules rather than basal bodies (Wang et al., 2013). Here you report a significant reduction in Cep290 signal at the basal body with mutated Cep162 in Drosophila (Fig. 3E). How can you explain this difference between human and fly?

**Fig. 5d and g from Wang et al., 2013**

**Answer：**In Wang et al., 2013, as illustrated in their Fig. 5d and g above, CEP290 was still recruited to the centriole/basal bodies in cells subjected to siRNA knockdown of CEP162. However, they did not quantify the signal intensity to determine whether it was reduced. It is important to note that this was a siRNA knockdown assay, and residual CEP162 may still be present. Consequently, further investigation is needed to ascertain whether the signal of CEP290 is indeed reduced in the absence of CEP162 in mammalian cells.

**3.** Could the quantification of the levels of GFP-fused proteins at the basal body be explained by differences in total expression levels of these GFP-fused proteins in wild-type and mutated Drosophila lines (Fig 1A, 1B, 2D, 3E, 4A, etc.). It's important to ensure that the reduction of these proteins in is not caused by protein expression.

**Answer：**We thank the reviewer for raising this critical concern.

For each GFP-fused protein transgenic line, in order to maintain consistent expression levels between WT and mutants, we initially created transgenic line by integrating the expression plasmid into the WT background using the PhiC31 integrase system. Subsequently, the transgene was crossed into the mutant background. Live imaging of GFP signals of Cby::GFP, Cep290-C::GFP, Cep290-N::GFP and MKS1::GFP in spermatocytes revealed that they all have a similar GFP intensity levels in the cytosol of WT and *cep131* or *cep162* mutants, supporting that they exhibit comparable expression levels. Representative images are showed in figure below.

In an effort to address the reviewer’s concern, we tried the western blot assay to detect the GFP protein levels. However, western blotting was challenging, likely influenced by both antibody performance in *Drosophila* and the lower expression level of basal body proteins. Then we conducted RT-PCR to assess the transcription level of GFP proteins, similar transcription level of GFP was observed in WT and *cep131* or *cep162* mutants. Representative images are presented in figure below.

Hence, there is no evidence to suggest that the observed reduction in signal in mutants is attributable to differences in expression levels.

**4.** The dynamic and temporal localization pattern of Cep131 and Cep162 during cilia development is interesting (Fig. 2E). Given that these two proteins also impact the levels of Cep290 on the basal body during the initiation of ciliogenesis, it raises the question of whether the levels of Cep290 at the cilia change during different stages of spermatocyte cilia formation. Evaluating this by quantifying the intensity of Cep290 at different stages of ciliogenesis in spermatocyte could provide more insight into this mechanism.

**Answer:** We appreciate the reviewer for this insightful question. As suggested by the reviewer, we investigated the Cep290 localization during spermatogenesis. As showed in the figure below, Cep290::GFP was not detectable on centriole in spermatogonia. During ciliogenesis in early spermatocytes, Cep290 appears at the tip of centriole, and the signal intensity increase in late spermatocytes and round spermatids. In late spermatids, Cep290 migrates with the ciliary cap, although the signal retained at the base of ciliary cap, its intensity reduced.

**5.** Given the differences in findings between human and fly it is important to be more specific about conclusions being made in the text. For example, description of findings to in the "Abstract".

**Answer:** In response to the suggestion, we have made revisions accordingly. *Drosophila* has been included in our abstract conclusion: “Here, we report that the Cep131-Cep162 module near the axoneme and the Cby-Fam92 module close to the membrane synergistically control the basal body localization of Cep290 and the subsequent initiation of ciliogenesis in *Drosophila*.”

**Rev. 2:**

CEP290 is a major structural element of the ciliary transition zone that was shown initially in Chlamydomonas to link the axoneme to the ciliary membrane. It has a central role in gating ciliary transport. Human CEP290 mutations are associated with a range of ciliopathy syndromes including but limited to MKS, JBTS, BBS, LCA, and SLS. This work builds on previous work showing that Cep290 integration into the transition zone requires Cep131 and Cby, and on prior work indicating that Cep290 associates with the axoneme through the C-terminal domain, and with the ciliary membrane through the N-terminal domain.

The authors provide strong evidence in Drosophila that Cep290 is integrated into the transition zone through association by its C-terminal region with a Cep131-Cep162 module at the axoneme, and with the ciliary membrane by its N-terminal region by association with the Cby-Fam92 module. They examine this molecular organization in spermatocyte cilia/spermatid flagella and in sensory cilia.

The authors show that Cep131 and Cep162 interact directly and generated deletion alleles of both genes. They additionally showed that Cep162 associates with Cep290 at its C-terminal region, providing a link between Cep290 and its association with the axoneme. They further show that the N-terminal region of Cep290 associates with the Cby-Fam92 module at the ciliary membrane. Mutation of either Cep131 or Cep162, together with either Cby or Fam92 produces a severe loss of cilia similar to the phenotype of loss of Cep290.

Overall, the work advances our understanding of how Cep290 integrates into the transition zone of cilia. A model depicting an epistatic ordering of the assembly of the components is supported by the findings and presented in Figure 7. The work is clearly communicated, and the experiments are well-presented and support the major findings of the work. **I have some minor points to address:**

We greatly appreciate the reviewer for these positive comments. Kindly find our responses addressing all of her/his points below.

**1.** For the deletion mutations generated for the cep131[1] mutant and the cep162[1] mutant, cep131[1] is described as a frameshift mutation in Supp Fig 1, and the same for cep162[1] in the legend for Figure 3. Please include the details in the Supp Figure 4. The authors should report what the predicted protein products are. The authors have the sequence data to discern this. For both mutants use standard nomenclature (eg "p.Arg131ProfsTer12"). This information could be included in Supp Figure 1 for cep131, and Supp Figure 4 for cep162.

**Answer:** Revised as suggested. We have added the information about the predicted protein products for Cep131^1^_p.(His480_Leu708delfsTer61) in Supplementary Fig. 1A, and Cep162^1^_p.(Glu327_Met436delfsTer24) in Fig. 3A.

**2.** The assembly of the long spermatid flagellum is unusual in that it is assembled in the cytoplasm while only the distal tip is encased in ciliary membrane, a ciliary cap, with a 'ring centriole' at its base. The authors show that in spermatocytes that are deficient in anchoring of centrioles to the membrane, the axoneme appears to overgrow. This is sometimes referred to in the manuscript as an axoneme and other times as 'abnormal extensions' that label with CG6655-GFP (Figures 5,6,S1,S4,), but in other contexts with Cep162, or Cep290 (Figure 4), indicating that they are something other than axonemes and maybe are also TZ-like. There are multiple figures where CG6655-GFP is used to label these axonemes/abnormal extensions as an apparent indication that the centriole did not anchor to the membrane. The authors should clarify what these 'abnormal extensions of the ciliary axonemes' are, or describe what they perceive them to be. Moreover, what is the consequence in spermatids

- do the axonemes grow despite lacking a ciliary cap?

**Answer:** In our manuscript, all abnormal signal labelled with CG6652-GFP (Fig. 5C, 6E, S1D, S4C) are **abnormal extensions of microtubules from giant centriole/basal body in spermatocytes**.

Given that these abnormal microtubule extensions form during the spermatocyte stage, while the cytosolic segment of spermatid flagella assembles in the spermatid stage, they should be different. In addition, since the mutants have defects in connecting centriole/basal body with the membrane, resulting in blocked ciliogenesis and the absence of cilia, it may not be appropriate to refer to these extensions as ciliary axoneme. We apologize for any confusion, and revision has been made accordingly in manuscript.

Current available data suggest that severe defects occur in the axoneme of flagella when lacking a ciliary cap. Sperm cysts were severely defective in elongation in *cby; cep131* double mutants (see below Fig. S6A from Vieillard et al., J Cell Biol. 2016) and *cep290* mutants (see below Fig. S1F from Wu et al., PLoS Biology, 2020). And TEM revealed that most axoneme were absent or damaged in *cby; cep131* double mutants (see below Fig. S3A from Vieillard et al., J Cell Biol. 2016), in *cep290* mutants (see below Fig. S1H from Wu et al., PLoS Biology, 2020), and *cby; cep162* double mutants (Fig. 5D, this paper). However, it is noteworthy that, despite severe defects, axoneme, at least partially, could still grow, as evidenced by the observation of a few intact axonemes (see Yellow arrows in EM images in figure below) in mutants.

**3.** In Figure 3D, TEM of the cep162 mutant is presented and shown to have fewer numbers of spermatids. Several of the spermatids are colorized but this is not described in the text or figure legend. The colorized ones appear to have very small mitochondrial derivatives, and it appears that overall the sizes of the mitochondrial derivatives are variable compared to WT. This feature of the mutant should be included in the analysis and discussed in the context of other publications describing links between cilium proteins and mitochondrial morphogenesis in Drosophila testis.

**Answer:** We appreciate the reviewer’s suggestion. We agree with the reviewer that mitochondrial abnormalities are indeed present in *cep162* mutants. As suggested, we have incorporated this information in the results section and figure legend. Kindly refer to our revised manuscript on page 9 to 10 for further details.

**4.** The legend for Figure 3 refers to a panel E with images showing the pattern of CG6652-GFP in the cep162 mutant, but this panel is missing. Panel E is described as F in the legend. The text also does not refer to these data or a panel F, so perhaps the legend info on the CG6652-GFP localization in the cep162 mutant spermatocytes should be deleted.

**Answer:** We apologize for this mistake. The legend of Figure 3, panel E, has been revised.

**5.** In Figure 4A, the cep162[1] cep290[1] double mutant is depicted in the graphic as having extended filaments, probably TZ/axonemes, presumably because they infer that the mutant centrioles do not anchor to the membrane, but no evidence is shown for their assembly. The authors can check whether CG6652-GFP labels distal filaments in the double mutant, or perhaps staining for acetylated tubulin will suffice to show these extensions. Also, in the legend for panel D, referring to ciliary signal for Cep290-C, the statement "Notably, Cep290-C::GFP signal is completely lost in cep162; cep2901 double mutants." should instead say 'significantly reduced'.

**Answer:** To address the reviewer’s concern, we stained the centriole/basal body with Ac-tub in *cep162;* *cep290^1^* double mutants, as illustrated in the figure below, confirming the presence of abnormal microtubule extensions in this double mutant. We have included this data in Supplementary Fig. 4E of revised manuscript.

Additionally, we have replaced the phrase “completely lost” with “significantly reduced”.

**6.** Figure 5: The absence of axonemes in the cep162; cby TEM images is difficult to interpret as there are no distinguishing feature. It might be more straightforward and informative to characterize the extent of axoneme assembly in these mutant spermatids by staining for acetylated tubulin (as was done in Figure 2E). It is worth deeper inspection because others have shown that spermatids can elongate even without centrioles (eg sak mutant spermatids), as elongation is driven by the mitochondrial derivative and does not require the axoneme. Perhaps these mutants are unexpectedly more severe than mutants lacking centrioles.

**Answer:** In *cep162; cby* double mutants, TEM analysis revealed severe defects in the axoneme of spermatid flagella. However, a few intact axonemes were still observed in *cep162; cby* double mutants (see Yellow arrows in EM image below), suggesting that partial spermatid axoneme can grow. This observation is consistent with previous reports in *cep290* mutant and *cby; cep131* double mutant. Additionally, upon examining flagella labeled with Ac-tub in round spermatids and early spermatids, we did observe assembled flagella (see Figure below) in *cpe162; cby* double mutants. Therefore, it is possible that the axoneme retains the ability to grow during the early stages of assembly but may lack stability.

**7.** In the Discussion, the authors propose that a ciliopathy phenotype might arise from double mutations in CEP131 or CEP162 with CBY1 or CIBAR1/2 (FAM92A/B). I assume part of this logic arises because no ciliopathies have been yet found associated with mutations in these genes, which is a point that should be stated. It is a worthwhile prediction, but it should just be pointed out that these genes are not yet clinically linked, while CEP290 is, and is likely attributed to the cooperative nature of these modules.

**Answer:** Revised as suggested. We mentioned in the last paragraph of the discussion that “While Cep131 and Cby has not yet been associated with ciliopathy, mutations in Cep162 and Fam92A have been reported to be linked to human cilia-related diseases”, and deleted the sentence “Furthermore, in ciliopathies with unknown disease gene, it is worth checking for the presence of the double mutations of Cep131-Cep162 module and Cby-Fam92 module.”.

**Additional points:**

Manuscript needs page numbers.

**Answer:** Revised. Page numbers have been added.

Please run a spellcheck on the ms, there are numerous typos.

Typos in Introduction: "strucutres", "Chibbly", "foramtion", "seemly", "cordinating", "stablize". In Results: "Collectivley". In Discussion: "accessbile". In legends: "Trpplates"

**Answer:** All have been revised. We sincerely apologize for our mistakes. We did a thorough the spellcheck and corrected all typos.
